# Effects of Basicity, MgO, and Al_2_O_3_ on Thermodynamic and Physicochemical Properties of CaO-SiO_2_-MgO-Al_2_O_3_ Slag System

**DOI:** 10.3390/molecules30214275

**Published:** 2025-11-03

**Authors:** Zicheng Xin, Jiangshan Zhang, Qing Liu

**Affiliations:** State Key Laboratory of Advanced Metallurgy, University of Science and Technology Beijing (USTB), Beijing 100083, China

**Keywords:** refining slag, thermodynamic simulation, property, CaO-SiO_2_-MgO-Al_2_O_3_ slag system, FactSage, KTH

## Abstract

Ladle furnace (LF) refining is one of the most widely used secondary refining processes for producing clean steel and constitutes a key process in the steelmaking–continuous casting section. The properties of slag play a decisive role in determining molten steel quality and refining efficiency. In this study, based on the composition of refining slag from a steelmaking plant in China, the properties of a CaO-SiO_2_-MgO-Al_2_O_3_ slag system were investigated with respect to five aspects, the liquid phase region, sulphide capacity, melting properties, slag viscosity, and mineralogical phase precipitation, at varying temperatures, basicity, *w*(MgO) and *w*(Al_2_O_3_) using FactSage and the KTH model. Analysis of the slag properties indicates that the CaO-SiO_2_-MgO-Al_2_O_3_ slag system performs better when basicity ranges from 3 to 4, *w*(MgO) is between 6% and 8%, and *w*(Al_2_O_3_) is 15%–25%. These findings provide theoretical support and guidance for optimizing the refining slag system in plant trials.

## 1. Introduction

In the ladle furnace (LF) refining process, slag plays a crucial role in deep deoxidization, deep desulfurization, submerged arc operation, absorption of inclusions, prevention of secondary oxidation of molten steel, and heat preservation of molten steel, thereby determining the high efficiency and stability of ladle furnace operations [1,2,3]. Numerous slag properties have been investigated by many researchers [4,5,6]. Kim et al. [7] investigated the effect of Al_2_O_3_ and CaO/SiO_2_ on the viscosity of the CaO-SiO_2_-10 mass pct MgO-Al_2_O_3_ slags at fully liquid temperatures of 1773 K (1500 °C) and below. Yao et al. [8] studied the effects of Al_2_O_3_, MgO, and CaO/SiO_2_ on the viscosity of high-alumina blast furnace slag at 1773 K (1500 °C) and below using the orthogonal experimental design method. Kim et al. [9] investigated the effect of MgO on the viscosity of CaO-SiO_2_-20%Al_2_O_3_-MgO slags at elevated temperatures by measuring the slag system’s viscosity.

Desulfurization plays a crucial role in the LF refining process. To quantify the desulfurization capacity of slag, Fincham and Richardson [10] proposed the concept of sulphide capacity (C_S_). Many C_S_ calculation models have been developed by metallurgists, among which optical basicity [11] and the KTH model [12] have been widely used for calculating the C_S_ of slag. Sosinsky et al. [13] investigated the relationship between C_S_ and optical basicity. Young et al. [11] optimized the C_S_ calculation model based on that of Sosinsky et al. Nzotta et al. [14] experimentally determined the C_S_ under different slag systems by means of experimental determination and developed the KTH model. The main idea of the KTH sulphide capacity model is to combine thermodynamic parameters with experimental data analysis to achieve an accurate prediction of sulphide capacity. According to Refs. [15,16,17], the C_S_ values calculated using the KTH model developed by the KTH Royal Institute of Technology (Stockholm, Sweden) are close to the actual values. However, most studies have focused on a single property, while few have systematically investigated the liquid phase region of slag, melting behaviour, and mineralogical phase precipitation. Due to time-consuming and costly high-temperature experiments, an experimental simulation was adopted in this study. FactSage [18,19,20,21] has been successfully used to calculate the thermodynamic data of metallurgical melts, providing valuable guidance for laboratory experimental design and plant trials. This approach not only achieves reliable experimental results but also reduces the experimental cost and risk.

In this study, based on the actual slag conditions at a steelmaking plant in China, the effect of basicity, MgO, and Al_2_O_3_ on the metallurgical properties of slag were investigated using FactSage, Photoshop, and the KTH model. Meanwhile, the precipitation behaviour of slag was simulated. Based on the simulation results, the effects of different factors on slag properties were further analyzed, providing theoretical guidance for the optimization of the slag system.

## 2. Calculation Methods

### 2.1. Slag Composition

Based on the composition of refining slag from a steelmaking plant in China, the basicity was designed to vary from 2.0 to 4.5 at 0.5 intervals. The MgO content was designed to vary from 0 to 10% at 2% intervals. The Al_2_O_3_ content was designed to vary from 10% to 35% at 5% intervals, as shown in Table 1.

### 2.2. Liquid Phase Region and Melting Properties

**Figure 1:**
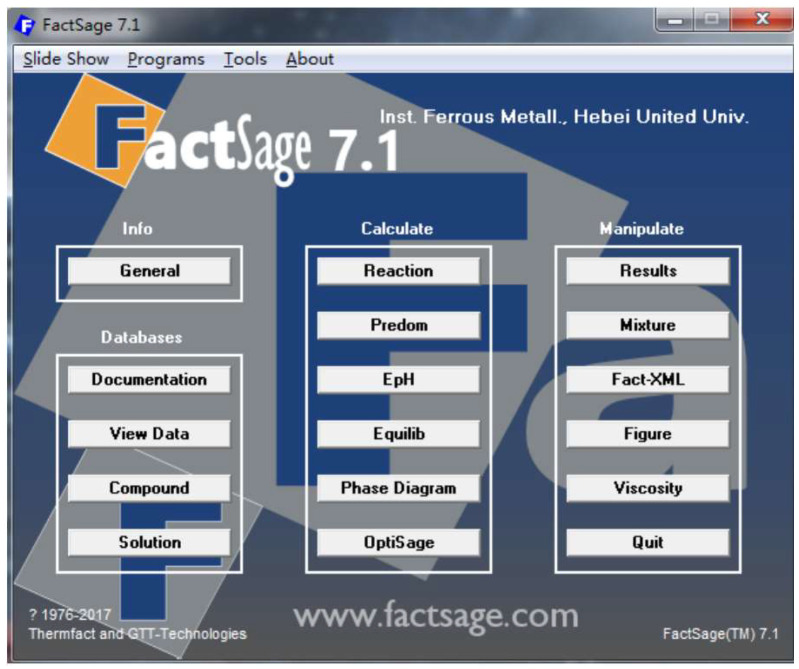
Interface of the FactSage7.1 software.

The effects of MgO and Al_2_O_3_ on the liquid phase region of the CaO-SiO_2_-MgO-Al_2_O_3_ slag system were calculated using the Phase module of the FactSage7.1 software, and the interface of the software is shown in Figure 1. The calculation steps are as follows:

Step 1: Select “FToxide” from the FactSage7.1 database and enter the components (CaO, SiO_2_, MgO, and Al_2_O_3_).

Step 2: Select “Solution Phases” by clicking the “Select” button and selecting all solution phases retrieved from the database.

Step 3: Set the parameters. The temperature ranges from 1300 °C to 1600 °C in increments of 100 °C. The MgO content ranges from 0 to 10% in increments of 2%. The Al_2_O_3_ content ranges from 10% to 35% in increments of 5%.

The effects of basicity, MgO, and Al_2_O_3_ on the melting properties and viscosity of the CaO-SiO_2_-MgO-Al_2_O_3_ slag system were calculated using the Equilib module and the Viscosity model of the FactSage7.1 software. The calculation steps are as follows:

Step 1: Select “FToxide” from the FactSage7.1 database; enter the CaO, SiO_2_, MgO, and Al_2_O_3_ components; and input their contents according to Table 1.

Step 2: Select “Solids” by clicking the “Select” button and selecting all possible solution phases retrieved from the database.

Step 3: If “IF” is selected from Ftoxid-Slag, the initial melting temperature of the slag will be calculated. If “IP” is chosen, the complete melting temperature will be calculated. The initial melting temperature refers to the temperature at which the liquid phase begins to form from the solid phase. The complete melting temperature refers to the temperature at which all solid phases in the system are completely transformed into the liquid phase.

### 2.3. Viscosity and Mineralogical Phase Precipitation

The viscosity calculation steps are as follows:

Step 1: Select “Melts” from the FactSage7.1 database.

Step 2: Enter the CaO, SiO_2_, MgO, and Al_2_O_3_ components; input their contents according to Table 1; and input the temperature.

Step 3: Use the Equilib module to determine whether the slag is completely in the liquid phase at the set temperature. If the slag is completely in the liquid phase, the viscosity is calculated using the Viscosity module. If not, the viscosity calculated using the Viscosity module is corrected using the Einstein–Roscoe equation [22,23], as shown in Equation (1).

Viscosity_(solid+liquid mixture)_ ≈ Viscosity_(liquid)_(1 − solid fraction)^−2.5^ (1)

The Sheil–Gulliver cooling method of the Equilib module was used to calculate mineralogical phase precipitation. The calculation steps are as follows:

Step 1: Select “FToxid” from the FactSage 7.1 database; enter the CaO, SiO_2_, MgO, and Al_2_O_3_ components; and input their contents according to Table 1.

Step 2: Set the temperature to 1600 °C to start cooling.

Step 3: Calculate the composition, precipitation temperature, and maximum precipitation amount of mineralogical phase precipitation using the Sheil–Gulliver cooling method.

### 2.4. Sulphide Capacity

The effects of basicity, MgO, and Al_2_O_3_ on the Cs of the CaO-SiO_2_-MgO-Al_2_O_3_ slag system were calculated using the KTH model.

The definition of C_S_ is as shown in Equation (2) [24].

(2) $$Cs=\frac{K\cdot a_{O^{2-}}}{f_{S^{2-}}}=(\%S)\sqrt{\frac{P_{O_{2}}}{P_{S_{2}}}}$$

where *K* is the equilibrium constant, $a_{O^{2-}}$ is the activity of ionic oxygen in slag, $f_{S^{2-}}$ is the activity of ionic sulphur in slag, (%S) is the weight percent of the sulphur content in slag, $P_{O_{2}}$ is the partial pressure of oxygen gas, and $P_{S_{2}}$ is the partial pressure of sulphur gas. Based on the definition of sulphide capacity in Equation (2), the calculation of sulphide capacity in the KTH model is expressed as (Equation (3)) [14].

(3) $$Cs=\exp\left\{-\frac{\Delta G^{\theta }}{RT}\right\}\cdot \left\{-\frac{a_{O^{2-}}}{f_{S^{2-}}}\right\}=\exp\left\{-\frac{\Delta G^{\theta }}{RT}\right\}\cdot \exp\left\{-\frac{\zeta }{RT}\right\}$$

where $\Delta G^{\theta }$ is the Gibbs energy (J/mol) and *R* is the gas constant, 8.314 J/mol·K. In the multi-component system, ζ is a function of the component and temperature, as shown in Equation (4).

(4) $$\zeta =\sum X_{i}\zeta_{i}+\zeta_{\text{mix}}$$

where *i* is a component, *X_i_* is the mole fraction of each component *i* in the multi-component system, $\sum X_{i}\zeta_{i}$ is a linear change in the interaction between the different components, and $\zeta_{\text{mix}}$ is the mixed interaction coefficient between components.

The calculation parameters of ζ are primarily obtained from Ref. [25]. The calculation of C_S_ is not the focus of this study; therefore, detailed calculation procedures are not discussed here.

## 3. Results and Discussion

### 3.1. Effects of Chemical Composition on the Liquid Phase Region

**Figure 2:**
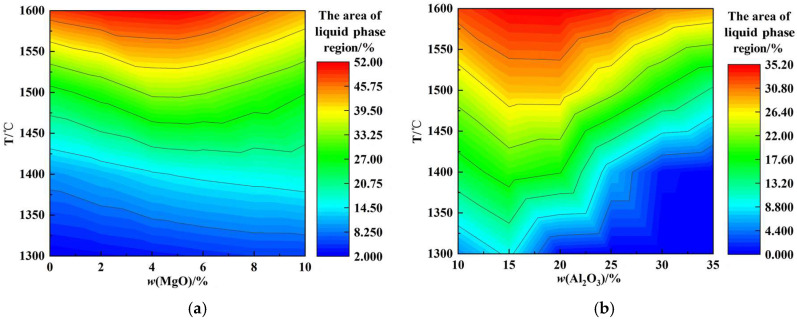
Effect of *w*(MgO) and *w*(Al_2_O_3_) on the liquid phase region (1300 °C–1600 °C): (**a**) *w*(MgO); (**b**) *w*(Al_2_O_3_).

Figure 2a shows the effects of *w*(MgO) and temperature on the liquid phase region of CaO-SiO_2_-MgO-Al_2_O_3_ slags. It is evident from Figure 2a that at fixed MgO values, the area of the liquid phase region increases with increasing temperature. Within the temperature range of 1300 °C to 1400 °C, the area of the liquid phase region increases. In the temperature range of 1500 °C–1600 °C, the area of the liquid phase region first increases and then decreases with increasing *w*(MgO) content, reaching a maximum at approximately 5% *w*(MgO). As *w*(MgO) increases, the proportion of free oxygen ions increases, modifying the aluminum–oxygen complex ionic clusters, $Al_{x}O_{y}^{z-}$, into small units that form low-melting-point compounds, thereby increasing the area of the liquid phase region. When *w*(MgO) further increases beyond its saturation percentage, the high-melting-point single-phase MgO solid solution grows, leading to a reduction in the liquid phase region [26,27].

Figure 2b shows the effects of *w*(Al_2_O_3_) and temperature on the liquid phase region of CaO-SiO_2_-MgO-Al_2_O_3_ slags. It is evident from Figure 2b that at fixed Al_2_O_3_ values, the area of the liquid phase region increases with increasing temperature. Within the temperature range of 1300 °C to 1400 °C, the area of the liquid phase region first increases and then decreases with increasing *w*(Al_2_O_3_) content, reaching a maximum at approximately 15% *w*(Al_2_O_3_). Similarly, in the range of 1500 °C to 1600 °C, the area of the liquid phase region first increases and then decreases as *w*(Al_2_O_3_) increases, reaching a maximum near 20% *w*(Al_2_O_3_). As *w*(Al_2_O_3_) increases, the maximum precipitation of high-melting-point single-phase solid solutions of MgO and CaO decreases, and the precipitation of high-melting-point silicates decreases, while the precipitation of low-melting-point aluminates increases, collectively expanding the liquid phase region. However, when *w*(Al_2_O_3_) exceeds a certain threshold, Ca_3_Al_2_O_6_ is transformed into CaAl_2_O_4_, and magnesium aluminum spinel forms, resulting in a reduction in the liquid phase region.

**Figure 3:**
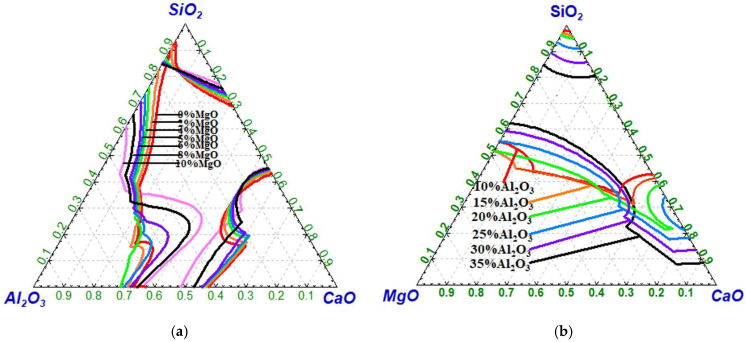
Effect of *w*(MgO) and *w*(Al_2_O_3_) on the liquid phase region (1600 °C): (**a**) *w*(MgO); (**b**) *w*(Al_2_O_3_).

Figure 3 illustrates the effects of *w*(MgO) and *w*(Al_2_O_3_) on the liquid phase region of CaO-SiO_2_-MgO-Al_2_O_3_ slags. Figure 3a illustrates the liquid phase regions of the CaO-SiO_2_-MgO-Al_2_O_3_ slag system with *w*(MgO) = 0, 2%, 4%, 6%, 8%, and 10%, respectively. Figure 3b shows the corresponding liquid phase regions of the CaO-SiO_2_-MgO-Al_2_O_3_ slag system with *w*(Al_2_O_3_) = 10%, 15%, 20%, 25%, 30%, and 35%, respectively. As shown in Figure 3a, at 1600 °C and fixed *w*(MgO) values of 0, 2%, 4%, 6%, 8%, and 10%, the CaO content at the boundary between the liquid-phase and non-liquid-phase zones—located near the lower part of the CaO-SiO_2_ axis—is approximately 56.8%, 56.0%, 55.4%, 55.1%, 54.7%, and 54.5%, respectively. The CaO content at this boundary decreases with increasing *w*(MgO) content. Figure 3b shows that the CaO content at the boundary between the liquid-phase and non-liquid-phase zones increases with increasing *w*(Al_2_O_3_) content [28].

### 3.2. Effects of Chemical Composition on the C_S_

**Figure 4:**
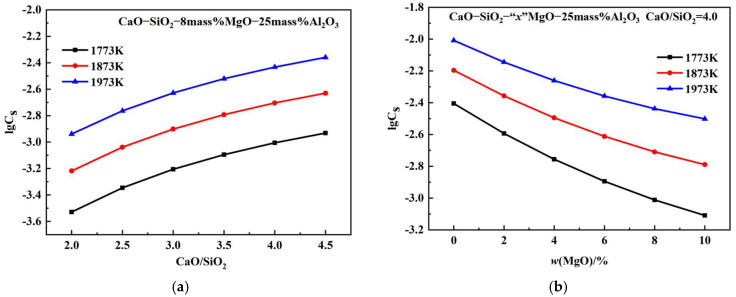

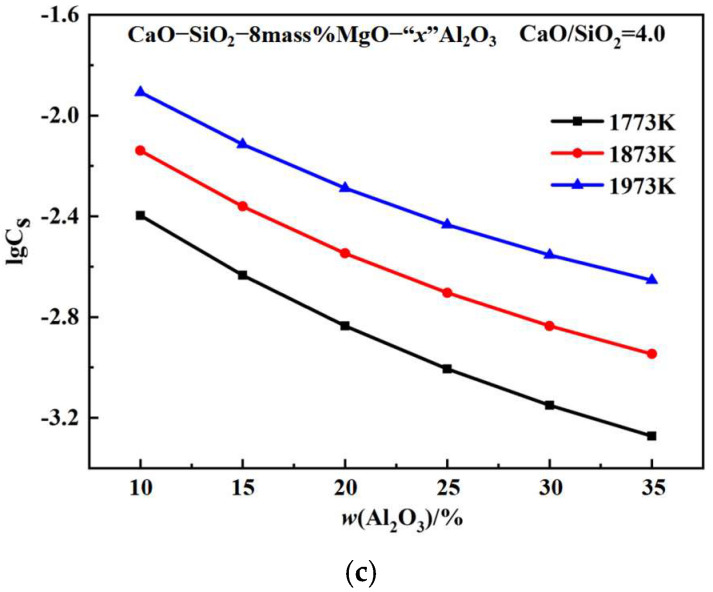
Effects of basicity, *w*(MgO), and *w*(Al_2_O_3_) on the sulphide capacity: (**a**) basicity; (**b**) *w*(MgO); (**c**) *w*(Al_2_O_3_).

Figure 4a shows the C_S_ of CaO-SiO_2_-MgO-Al_2_O_3_ slags with a *w*(MgO) value of 8% and a *w*(Al_2_O_3_) of 25% at temperatures of 1773 K, 1873 K, and 1973 K, plotted as a function of basicity. At fixed 8% MgO, 25% Al_2_O_3_, and temperatures, the sulphide capacity increases with increasing basicity. At a fixed slag composition, the sulphide capacity increases with increasing temperature. As the *w*(CaO) increases, the SiO_2_ networks simplify into smaller anion groups, and the proportion of free oxygen ions increases, increasing the sulphide capacity [29]. Figure 4b shows the effect of *w*(MgO) on the C_S_ of CaO-SiO_2_-MgO-Al_2_O_3_ slags with 25% Al_2_O_3_ and CaO/SiO_2_ at a ratio of 4.0. The sulphide capacity decreases with increasing *w*(MgO) content. The equilibrium constant for the reaction between CaO and S_2_ is higher than that between MgO and S_2_. As *w*(MgO) increases, CaO is partially replaced by MgO, reducing the saturation solubility of CaO and thus decreasing the C_S_ [30]. Figure 4c shows the effect of *w*(Al_2_O_3_) on the C_S_ of CaO-SiO_2_-MgO-Al_2_O_3_ slags with 8% MgO and CaO/SiO_2_ at a ratio of 4.0. The sulphide capacity decrease with increasing *w*(Al_2_O_3_) content. Al_2_O_3_ is an amphoteric oxide that readily combines with free oxygen ions to form aluminum–oxygen composite ions in basic slag, thereby reducing free oxygen ions and decreasing the C_S_ [31].

### 3.3. Effects of Chemical Composition on the Melting Properties

**Figure 5:**
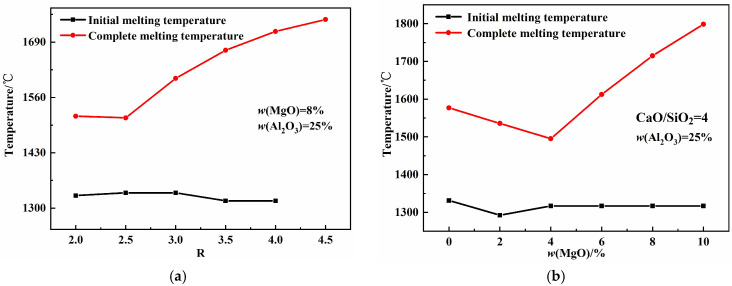

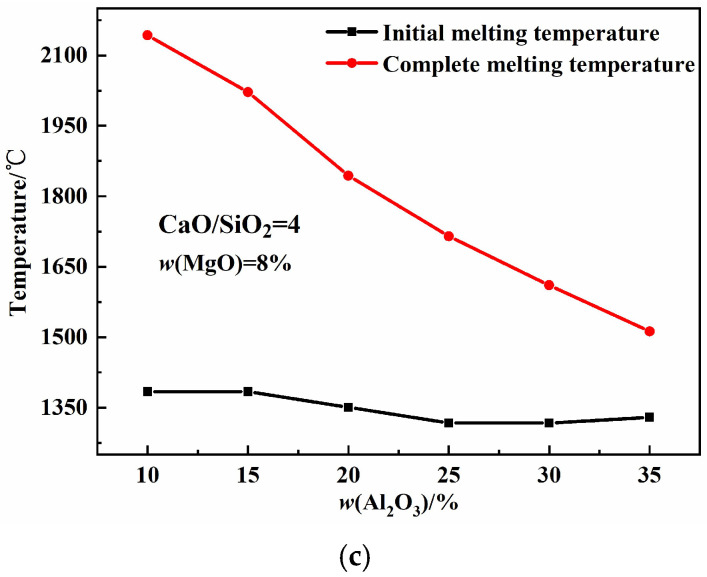
Effects of basicity, *w*(MgO), and *w*(Al_2_O_3_) on melting properties: (**a**) basicity; (**b**) *w*(MgO); (**c**) *w*(Al_2_O_3_).

Figure 5 illustrates the effects of basicity, *w*(MgO), and *w*(Al_2_O_3_) on the melting properties of the CaO-SiO_2_-MgO-Al_2_O_3_ slag system. As shown in Figure 5a, the initial melting temperature first increases and then decreases with increasing basicity, while the complete melting temperature increases. The increase in basicity promotes the formation of high-melting-point single-phase CaO solid solutions, leading to the rise in complete melting temperature. Figure 5b shows that both the initial and complete melting temperatures first decrease and then increase with increasing *w*(MgO) content. As the *w*(MgO) values increase, the proportion of free oxygen ions increases, and the aluminum–oxygen complex ionic clusters $Al_{x}O_{y}^{z-}$ break down into smaller units, forming low-melting-point compounds that reduce the complete melting temperature. When *w*(MgO) exceeds its saturation percentage, the high-melting-point single-phase MgO solid solution increases, causing the complete melting temperature to rise [26,27]. Figure 5c shows that the initial melting temperature first decreases and then increases with increasing *w*(Al_2_O_3_), whereas the complete melting temperature decreases continuously. Mineralogical phase precipitation indicates that increasing the *w*(Al_2_O_3_) content reduces the maximum precipitation of high-melting-point single-phase MgO and CaO solid solutions, as well as high-melting-point silicates, while increasing the precipitation of low-melting-point aluminates, collectively lowering the complete melting temperature.

### 3.4. Effects of Chemical Composition on Viscosity

**Figure 6:**
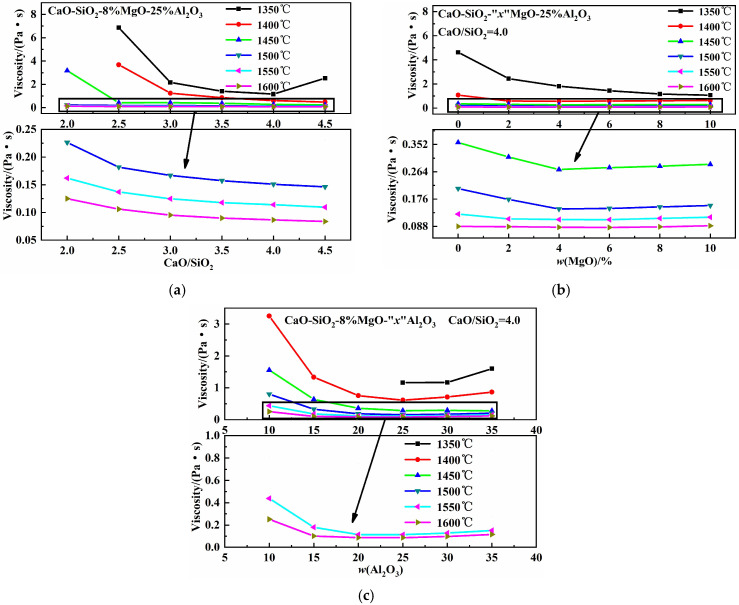
Effects of basicity, *w*(MgO), and *w*(Al_2_O_3_) on viscosity: (**a**) basicity; (**b**) *w*(MgO); (**c**) *w*(Al_2_O_3_).

Figure 6 illustrates the effects of basicity, *w*(MgO), and *w*(Al_2_O_3_) on the viscosity of the CaO-SiO_2_-MgO-Al_2_O_3_ slag system. As shown in Figure 6a, at 1350 °C, viscosity first decreases and then increases with increasing basicity, reaching a minimum at a basicity value of four. When basicity increases to a certain degree, the high-melting-point compounds form and the inhomogeneous phase content increases, causing viscosity to rise [32]. Between 1400 °C and 1600 °C, the slag’s viscosity decreases with increasing basicity. According to the ionic structure theory of liquid metallurgical slags [33], increasing basicity raises *w*(CaO), the proportion of free oxygen ions (O^2−^), and the O/Si ratio. This simplifies complex silicon–oxygen composite anions into simpler ones, reducing viscosity. Figure 6b shows that at 1350 °C, viscosity decreases with increasing *w*(MgO) content. Between 1400 °C and 1500 °C, viscosity first decreases and then increases with increasing *w*(MgO) content, reaching a minimum at 4% *w*(MgO). Between 1550 °C and 1600 °C, a similar trend is observed, with viscosity minimizing at 6% *w*(MgO). MgO, as a basic oxide, supplies free oxygen ions. Increasing *w*(MgO) raises the free oxygen ion content and breaks down aluminum–oxygen complex ionic clusters, $Al_{x}O_{y}^{z-}$, into smaller units, forming low-melting-point compounds and reducing viscosity [26]. Further increases in *w*(MgO) promote the formation of high-melting-point MgO single-phase solid solutions, leading to higher viscosity [27,34]. According to production experience at a Chinese steelmaking plant, slag causes minimal refractory erosion when *w*(MgO) is around 8%. Figure 6c shows that between 1400 °C and 1600 °C, viscosity first decreases and then increases with increasing *w*(Al_2_O_3_) content, reaching a minimum at 25% *w*(Al_2_O_3_). As the *w*(Al_2_O_3_) content increases, it promotes the transformation of high-melting 2CaO·SiO_2_ into low-melting calcium alumina feldspar [35]. When *w*(Al_2_O_3_) exceeds 25%, abundant complex aluminum–oxygen composite anions form, and mineralogical phase precipitation indicates that Ca_3_Al_2_O_6_ transforms into CaAl_2_O_4_, causing viscosity to increase [26].

### 3.5. Effects of Chemical Composition on Mineralogical Phase Precipitation

**Figure 7:**
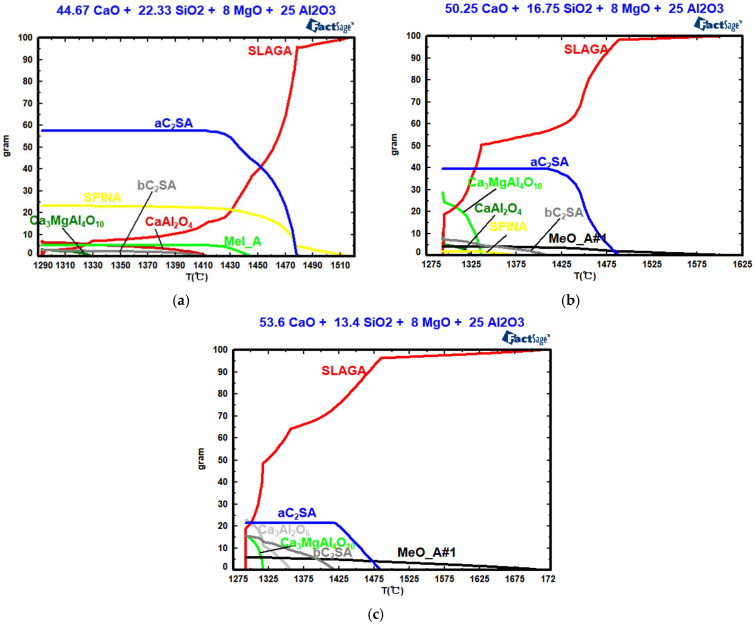
Effects of basicity on the mineral composition: (**a**) R = 2; (**b**) R = 3; (**c**) R = 4.

Figure 7, Table 2 and Table 3 show the effects of basicity on mineral composition, the crystal phase’s precipitation temperature, and the amount of crystal phase precipitation. As shown in Figure 7, when the basicity values are two, three, and four, the silicate (a-Ca_2_SiO_4_) begins to precipitate at 1478 °C, 1488 °C, and 1485 °C, respectively. With increasing basicity, the precipitation temperature first increases and then decreases, while the maximum precipitation decreases. When the basicity values are two, three, and four, the CaO and MgO solid solution (MeO_A#1) begins to precipitate at 1445 °C, 1605 °C, and 1715 °C, respectively. Increasing basicity raises the precipitation temperature of MeO_A#1, while the maximum precipitation of MeO_A#1 first decreases and then increases. When the basicity values are two and three, the magnesium aluminate spinel (SPINA) begins to precipitate at 1516 °C and 1376 °C. With increasing basicity, the precipitation temperature increases, whereas the maximum precipitation decreases. When the basicity values are two, three, and four, the silicate (b-Ca_2_SiO_4_) begins to precipitate at 1413 °C, 1410 °C, and 1419 °C, respectively. With increasing basicity, the maximum precipitation of silicate increases. When the basicity values are two and three, the calcium–aluminum spinel (CaAl_2_O_4_) begins to precipitate at 1413 °C and 1326 °C. When the basicity value is four, tricalcium aluminate (Ca_3_Al_2_O_6_) begins to precipitate at 1357 °C. When the basicity values are two, three, and four, the Ca_3_MgAl_4_O_10_ begins to precipitate at 1330 °C, 1336 °C, and 1317 °C, respectively. When the basicity value increases from 2 to 3, CaAl_2_O_4_ begins to precipitate, and its maximum precipitation increases. At a basicity value of four, Ca_3_Al_2_O_6_ begins to precipitate. With increasing basicity, the precipitation temperature and the maximum precipitation of Ca_3_MgAl_4_O_10_ first increase and then decrease; meanwhile, the maximum precipitation of silicates decreases, and the maximum precipitation of aluminate increases. The solidification endpoint of the CaO-SiO_2_-8% MgO-25% Al_2_O_3_ slag system remains constant at 1293 °C across various basicity values.

**Figure 8:**
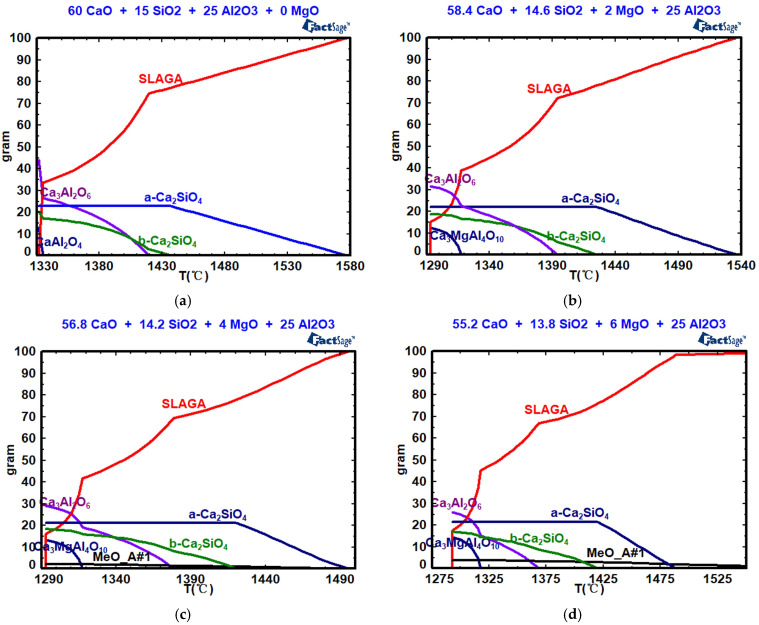

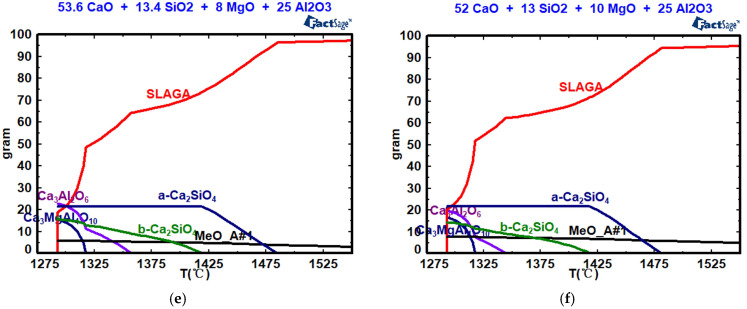
Effects of *w*(MgO) on the mineral composition: (**a**) *w*(MgO) = 0; (**b**) *w*(MgO) = 2%; (**c**) *w*(MgO) = 4%; (**d**) *w*(MgO) = 6%; (**e**) *w*(MgO) = 8%; (**f**) *w*(MgO) = 10%.

Figure 8, Table 4 and Table 5 show the effects of *w*(MgO) on mineral composition, the precipitation temperature of the crystal phase, and the amount of crystal phase precipitation. As shown in Figure 8, when the *w*(MgO) values are 0, 2%, 4%, 6%, 8%, and 10%, the silicate (a-Ca_2_SiO_4_) begin to precipitate at 1577 °C, 1536 °C, 1495 °C, 1488 °C, 1485 °C, and 1481 °C, respectively. With increasing *w*(MgO) content, both the precipitation temperature and the maximum precipitation decrease. When the *w*(MgO) values are 0, 2%, 4%, 6%, 8%, and 10%, the silicate (b-Ca_2_SiO_4_) begin to precipitate at 1437 °C, 1425 °C, 1420 °C, 1420 °C, 1419 °C, and 1418 °C, respectively. With increasing *w*(MgO) content, both the precipitation temperature and the maximum precipitation decrease. When the *w*(MgO) values are 0, 2%, 4%, 6%, 8%, and 10%, tricalcium aluminate (Ca_3_Al_2_O_6_) begins to precipitate at 1419 °C, 1393 °C, 1378 °C, 1368 °C, 1357 °C, and 1344 °C, respectively. With increasing *w*(MgO) content, both the precipitation temperature and the maximum precipitation of tricalcium aluminate decrease. When the *w*(MgO) values are 2%, 4%, 6%, 8%, and 10%, Ca_3_MgAl_4_O_10_ begins to precipitate at 1317 °C. Increasing the *w*(MgO) content does not affect the precipitation temperature, but it increases the maximum precipitation of Ca_3_MgAl_4_O_10_. The solidification endpoint of the CaO-SiO_2_-Al_2_O_3_ slag system is 1332 °C. For the CaO-SiO_2_-“*x*” MgO-25% Al_2_O_3_ slag system, the solidification endpoint remains at 1293 °C, regardless of the *w*(MgO) content. The content of the high-melting-point MgO single-phase solid solution increases from 1.92% to 7.52% as *w*(MgO) increases from 4% to 10%, resulting in an increase in viscosity.

**Figure 9:**
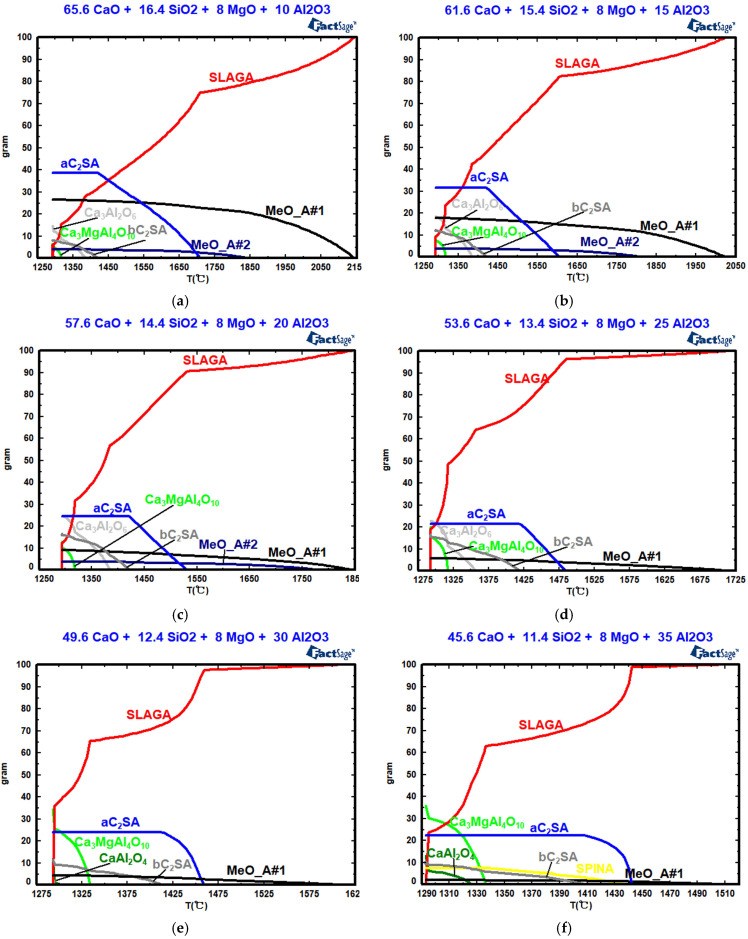
Effects of *w*(Al_2_O_3_) on the mineral composition: (**a**) *w*(Al_2_O_3_) = 10%; (**b**) *w*(Al_2_O_3_) = 15%; (**c**) *w*(Al_2_O_3_) = 20%; (**d**) *w*(Al_2_O_3_) = 25%; (**e**) *w*(Al_2_O_3_) = 30%; (**f**) *w*(Al_2_O_3_) = 35%.

Figure 9, Table 6 and Table 7 show the effects of *w*(Al_2_O_3_) on the mineral composition, the precipitation temperature of the crystal phase, and the amount of crystal phase precipitation. In Figure 9, as *w*(Al_2_O_3_) increases, the precipitation temperature and maximum precipitations of the solid solutions (MeO_A#1 and MeO_A#2) and silicates decrease during slag cooling, significantly enhancing slag fluidity. With increasing *w*(Al_2_O_3_) content, the precipitation temperature of tricalcium aluminate (Ca_3_Al_2_O_6_) decreases, while its maximum precipitation increases. However, with increasing *w*(Al_2_O_3_) content, the precipitation temperature and the maximum precipitation of CaAl_2_O_4_ and Ca_3_MgAl_4_O_10_ increase. Ca_3_Al_2_O_6_ begins to precipitate, and its maximum precipitation increases as *w*(Al_2_O_3_) increases from 10% to 25%. When *w*(Al_2_O_3_) exceed 25%, Ca_3_Al_2_O_6_ transforms into CaAl_2_O_4_. In actual production, an appropriate *w*(Al_2_O_3_) value promotes the formation of a low-melting-point phase, thereby reducing the slag melting point and viscosity [36]. The solidification endpoint of the CaO-SiO_2_-8%MgO-“*x*” Al_2_O_3_ slag system remains at 1293 °C across different *w*(Al_2_O_3_) values. In future work, high-temperature experiments and plant trials will be conducted to further validate the results.

## 4. Conclusions

The properties of the CaO-SiO_2_-MgO-Al_2_O_3_ slag system were evaluated from five aspects: the liquid phase region, sulphide capacity, melting behaviour, viscosity, and mineral phase precipitation.

(1) The liquid phase region (1300 °C–1400 °C) expands with increasing temperature and MgO content. It reaches a maximum at *w*(Al_2_O_3_) = 15% or at *w*(MgO) = 5% with *w*(Al_2_O_3_) = 20%, then decreases with further additions.
(2) Sulphide capacity decreases with increasing MgO and Al_2_O_3_ contents, but this can be mitigated with higher basicity and temperatures. The initial melting temperature is relatively insensitive to composition changes, whereas the complete melting point rises with basicity and decreases with added Al_2_O_3_, reaching a minimum at w(MgO) = 4%.
(3) Slag viscosity exhibits a complex dependence on temperature and composition. At 1300 °C–1400 °C, viscosity decreases with increasing MgO content and reaches a minimum at basicity = 4. At 1550 °C–1600 °C, viscosity decreases with increasing basicity and is lowest at *w*(MgO) = 6%. Across 1400 °C–1600 °C, viscosity is lowest at *w*(Al_2_O_3_) = 25%.
(4) Basicity and oxide contents significantly affect mineral phase precipitation. With increasing basicity, the maximum precipitation of Ca_3_MgAl_4_O_10_ first increases and then decreases. The MgO and Ca_3_MgAl_4_O_10_ solid solution’s contents increase with increasing *w*(MgO) content. Higher Al_2_O_3_ content promotes the precipitation of CaAl_2_O_4_ and Ca_3_MgAl_4_O_10_; when *w*(Al_2_O_3_) > 25%, Ca_3_Al_2_O_6_ transforms into CaAl_2_O_4_. Overall, optimal slag properties are obtained at basicity = 3–4, *w*(MgO) = 6%–8%, and *w*(Al_2_O_3_) = 15%–25%, offering theoretical guidance for optimization of the refining slag system.

## Figures and Tables

**Table 1:** Composition of refining slag.

Experiment Series	Component	Content/%					
A: Basicity variation	CaO	44.67	47.86	50.25	52.11	53.60	54.82
	SiO_2_	22.33	19.14	16.75	14.89	13.40	12.18
	MgO	8.00	8.00	8.00	8.00	8.00	8.00
	Al_2_O_3_	25.00	25.00	25.00	25.00	25.00	25.00
B: MgO content variation	CaO	60	58.4	56.8	55.2	53.6	52
	SiO_2_	15	14.6	14.2	13.8	13.4	13
	MgO	0	2	4	6	8	10
	Al_2_O_3_	25	25	25	25	25	25
C: Al_2_O_3_ content variation	CaO	65.6	61.6	57.6	53.6	49.6	45.6
	SiO_2_	16.4	15.4	14.4	13.4	12.4	11.4
	MgO	8	8	8	8	8	8
	Al_2_O_3_	10	15	20	25	30	35

**Table 2:** Effect of basicity on the precipitation temperature of the crystal phase (°C).

No.	a-Ca_2_SiO_4_	b-Ca_2_SiO_4_	CaAl_2_O_4_	Ca_3_Al_2_O_6_	Ca_3_MgAl_4_O_10_	MeO_A#1	SPINA
R = 2	1478	1413	1413	-	1330	1445	1516
R = 3	1488	1410	1326	-	1336	1605	1376
R = 4	1485	1419	-	1357	1317	1715	-

**Table 3:** Effect of basicity on crystal phase precipitation (%).

No.	a-Ca_2_SiO_4_	b-Ca_2_SiO_4_	CaAl_2_O_4_	Ca_3_Al_2_O_6_	Ca_3_MgAl_4_O_10_	MeO_A#1	SPINA
R = 2	57.39	2.71	7.12	-	3.46	5.05	22.98
R = 3	39.51	8.19	10.46	-	28.68	3.91	1.71
R = 4	21.54	15.56	-	22.83	15.56	5.67	-

**Table 4:** Effect of *w*(MgO) content on the precipitation temperature of the crystal phase (°C).

No.	a-Ca_2_SiO_4_	b-Ca_2_SiO_4_	Ca_3_Al_2_O_6_	Ca_3_MgAl_4_O_10_	MeO_A#1	CaAl_2_O_4_
MgO-0	1577	1437	1419	-	-	1335
MgO-2	1536	1425	1393	1317	-	-
MgO-4	1495	1420	1378	1317	1480	-
MgO-6	1488	1420	1368	1317	1550	-
MgO-8	1485	1419	1357	1317	1550	-
MgO-10	1481	1418	1344	1317	1550	-

**Table 5:** Effect of *w*(MgO) on crystal phase precipitation (%).

No.	a-Ca_2_SiO_4_	b-Ca_2_SiO_4_	Ca_3_Al_2_O_6_	Ca_3_MgAl_4_O_10_	MeO_A#1	CaAl_2_O_4_
MgO-0	22.71	20.29	43.97	-	-	13.03
MgO-2	22.10	18.72	31.54	12.45	-	-
MgO-4	21.26	18.30	28.97	13.37	1.92	-
MgO-6	21.41	16.92	25.90	14.47	3.79	-
MgO-8	21.54	16.71	22.83	15.57	5.65	-
MgO-10	21.63	14.24	19.76	21.40	7.52	-

**Table 6:** Effect of *w*(Al_2_O_3_) on the precipitation temperature of the crystal phase (°C).

No.	a-Ca_2_SiO_4_	b-Ca_2_SiO_4_	Ca_3_Al_2_O_6_	CaAl_2_O_4_	Ca_3_MgAl_4_O_10_	MeO_A#1	MeO_A#2	SPINA
Al_2_O_3_-10	1707	1421	1384	-	1317	2143	1846	-
Al_2_O_3_-15	1604	1421	1384	-	1317	2022	1809	-
Al_2_O_3_-20	1530	1421	1384	-	1317	1844	1779	-
Al_2_O_3_-25	1485	1419	1357	-	1317	1715	-	-
Al_2_O_3_-30	1459	1413	-	1302.7	1334	1610	-	-
Al_2_O_3_-35	1442	1409	-	1325.8	1336	1512	-	1437

**Table 7:** Effect of *w*(Al_2_O_3_) on crystal phase precipitation (%).

No.	a-Ca_2_SiO_4_	b-Ca_2_SiO_4_	Ca_3_Al_2_O_6_	CaAl_2_O_4_	Ca_3_MgAl_4_O_10_	MeO_A#1	MeO_A#2	SPINA
Al_2_O_3_-10	38.56	8.26	14.71	-	6.45	26.38	3.85	-
Al_2_O_3_-15	31.53	12.45	18.42	-	9.71	17.68	3.77	-
Al_2_O_3_-20	24.47	16.65	24.64	-	13.00	8.90	3.71	-
Al_2_O_3_-25	21.54	16.71	22.83	-	19.98	5.67	-	-
Al_2_O_3_-30	23.92	11.43	-	11.64	34.50	4.13	-	-
Al_2_O_3_-35	22.12	10.34	-	13.07	35.83	1.84	-	7.40

## Data Availability

The data presented in this study are available on request from the corresponding author due to privacy.
